# How do Uremic Toxins Affect the Endothelium?

**DOI:** 10.3390/toxins12060412

**Published:** 2020-06-20

**Authors:** Regiane Stafim da Cunha, Andressa Flores Santos, Fellype Carvalho Barreto, Andréa Emilia Marques Stinghen

**Affiliations:** 1 Experimental Nephrology Laboratory, Basic Pathology Department, Universidade Federal do Paraná, Curitiba 81531-980, Brazil; regidacunha@gmail.com (R.S.d.C.); andressaflores@ufpr.br (A.F.S.); 2 Internal Medicine Department, Division of Nephrology, Universidade Federal do Paraná, Curitiba 80060-900, Brazil; fellype.barreto@ufpr.br

**Keywords:** uremic toxins, endothelium, endothelial dysfunction

## Abstract

Uremic toxins can induce endothelial dysfunction in patients with chronic kidney disease (CKD). Indeed, the structure of the endothelial monolayer is damaged in CKD, and studies have shown that the uremic toxins contribute to the loss of cell–cell junctions, increasing permeability. Membrane proteins, such as transporters and receptors, can mediate the interaction between uremic toxins and endothelial cells. In these cells, uremic toxins induce oxidative stress and activation of signaling pathways, including the aryl hydrocarbon receptor (AhR), nuclear factor kappa B (NF-κB), and mitogen-activated protein kinase (MAPK) pathways. The activation of these pathways leads to overexpression of proinflammatory (e.g., monocyte chemoattractant protein-1, E-selectin) and prothrombotic (e.g., tissue factor) proteins. Uremic toxins also induce the formation of endothelial microparticles (EMPs), which can lead to the activation and dysfunction of other cells, and modulate the expression of microRNAs that have an important role in the regulation of cellular processes. The resulting endothelial dysfunction contributes to the pathogenesis of cardiovascular diseases, such as atherosclerosis and thrombotic events. Therefore, uremic toxins as well as the pathways they modulated may be potential targets for therapies in order to improve treatment for patients with CKD.

## 1. Introduction

Chronic kidney disease (CKD) is caused by progressive loss of kidney function, leading to the accumulation of uremic toxins in the bloodstream; the endothelium, which is directly exposed to these toxins, is negatively affected. The endothelium plays an important role in the maintenance of vascular homeostasis and its dysfunction may contribute to the pathogenesis of cardiovascular diseases (CVD) [1,2]. Clinical studies have shown that CKD patients are at increased risk of morbidity and mortality from CVD, such as atherosclerosis and thrombotic events [1,3,4,5,6,7]. In fact, multiple studies indicate that uremic toxins mediate endothelial dysfunction and vascular inflammation, both main contributors to the development of CVD [1,2].

Endothelial cells are exposed to a variety of uremic toxins, which can be divided into three major groups: (I) small water-soluble compounds; (II) middle molecules; and (III) protein-bound uremic toxins [8,9]. Small water-soluble compounds have a molecular weight of less than 500 Da, such as urea, uric acid, and guanidines [8,10]. The middle molecules have a molecular weight greater than 500 Da, among which are leptin, adiponectin, and β2-microglobulin [8,10,11,12]. Finally, protein-bound uremic toxins generally have low molecular weight and are difficult to remove by dialysis therapies. Prototypes of this group are *p*-cresyl sulfate (PCS), indoxyl sulfate (IS), and indole-3 acetic acid (IAA) [3,8,10].

**Figure 1:**
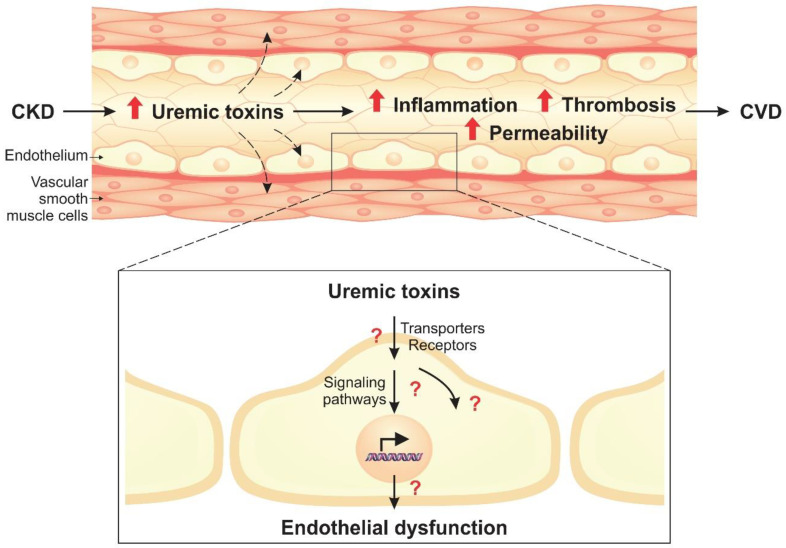
The progression of chronic kidney disease (CKD) results in the accumulation of uremic toxins in the bloodstream, which leads to endothelial dysfunction. Vascular endothelial cells exposed to uremic toxins have a proinflammatory and a prothrombotic phenotype, and the monolayer structure is damaged, increasing permeability. This impairment of endothelial function can contribute to cardiovascular diseases (CVD) pathogenesis.

Recently, in vivo and in vitro studies have investigated the effects of isolated uremic toxins or uremic serum from CKD patients on endothelial cells to better understand the molecular and cellular mechanisms involved in endothelial dysfunction mediated by uremic toxins [13,14]. This review aims to discuss the main findings of the impact of uremic toxins on the endothelium, including effects on cell structure, expression of receptors and transporters of uremic toxins, activation of signaling pathways, microRNA (miRNA) regulation, inflammation and thrombosis processes, and formation of endothelial microparticles (EMPs) (Figure 1).

## 2. Endothelial Dysfunction in CKD

In CKD, impairment of endothelial function is characterized by increased oxidative stress, expression of proinflammatory and prothrombotic molecules, structural damage, and failure of the endothelial repair and protection mechanisms. Uremic toxins can contribute to these deleterious effects on the endothelium, as demonstrated by several in vitro and in vivo studies. Although this review addresses the effects of uremic toxins on the endothelium, it is important to keep in mind that other factors also contribute to endothelial dysfunction in CKD patients, such as shear stress. In fact, laminar shear stress is a hemodynamic force with a relevant role in maintaining endothelial functions, including the production of nitric oxide (NO), vasodilation, and permeability [15,16]. However, patients with end-stage renal disease (ESRD) have low shear stress, which is associated with increased EMPs and vascular dysfunction [17,18,19,20].

### 2.1. Uremic Toxins Induce Inflammation and Oxidative Stress in Endothelial Cells

CKD patients develop CVD and inflammation, especially in advanced stages of the disease, as the regulatory properties of the vascular endothelium are altered leading to diapedesis and immune cell activity [21,22,23,24]. In response to the injury caused by uremic toxins to the endothelium, the concentration of proinflammatory cytokines and chemokines in the bloodstream, such as monocyte chemoattractant protein-1 (MCP-1), vascular endothelial growth factor (VEGF), stromal cell-derived factor-1 (SDF-1), tumor necrosis factor alpha (TNF-α), interleukin-1 beta (IL-1β), interleukin-6 (IL-6), and high sensitivity C-reactive protein (hsCRP), is altered [1,22,23,25,26,27]. During the inflammatory process, MCP-1 attracts monocytes and macrophages to the injured endothelium. We have previously demonstrated that MCP-1 expression increases with higher concentrations of the toxins (IS and PCS) in the human endothelial cell line (EA.hy926) [14], and *p*-cresol (PC) and PCS on vascular smooth muscle cells (VSMC), via nuclear factor kappa B (NF-κB) [28]. We also demonstrated the presence of elevated levels of MCP-1 in uremic plasma, especially in patients with advanced stage of CKD [29]. VEGF and SDF-1 attract endothelial progenitor cells (EPCs) from the bone marrow to the injury site, leading to the activation of endothelial cells [22,25,26,30]. Ribeiro et al. [30] showed that uremic serum can decrease the levels of SDF-1 in human umbilical vein endothelial cells (HUVECs) in comparison with healthy serum after 6 h, while IL-8 concentration increased within 12 h, indicating a poor vascular adaptation of patients with CKD [31,32].

Uremic toxins also alter the expression of adhesion molecules, such as E-selectin, P-selectin, intercellular adhesion molecule-1 (ICAM-1), and vascular cell adhesion molecule-1 (VCAM-1), which promote the infiltration of monocytes and macrophages in the activated endothelium [22,23,25,26]. In fact, a positive correlation between IS, PCS, and IAA in the pre-dialysis plasma of CKD patients and soluble vascular cell adhesion molecule-1 (sVCAM-1) has been observed [27]. Shen et al. [33] demonstrated that IS increased IL-1β-induced E-selectin expression and monocyte adhesion in HUVECs. Ito et al. [34] also showed that IS enhanced E-selectin expression in TNF-α-treated HUVECs. The regulation of E-selectin expression involves several intracellular signaling pathways, including phosphorylation of mitogen-activated protein kinases (MAPKs), activation of NADPH oxidase, reactive oxygen species (ROS) production, and NF-κB signaling, as well as aryl hydrocarbon receptor (AhR) [26,33,34]. Six et al. [35] used in vitro aortic rings of wild type mice to show that IS can increase the expression of ICAM-1 and VCAM-1. Hyperphosphatemia also enhanced the expression of ICAM-1 and VCAM-1 in the aortic endothelium of CKD mice [36]. Similarly, Jing et al. [1] demonstrated that PCS induced the expression of ICAM-1, VCAM-1, and E-selectin in HUVECs, as well as enhancing monocyte–endothelium interaction in vitro and in vivo. Interestingly, the authors observed a greater amount of atherosclerotic lesions and macrophage infiltration in the aortas of nephrectomized mice treated with PCS compared to the sham group, indicating that PCS induces atherogenesis [1]. Hippurate, another uremic toxin, can also increase the expression of ICAM-1 and decrease the production of endothelial nitric oxide synthase (eNOS) in human aortic endothelial cells (HAECs), contributing to cellular dysfunction [37].

In vitro and in vivo studies have shown that uremic toxins lead to inhibition or reduction of NO production, which is an important regulator of vascular tone, while promoting the production of ROS, resulting in oxidative stress [38,39,40]. Uremic toxins can also decrease antioxidants defenses, such as superoxide dismutase (SOD), catalase, glutathione peroxidase, and glutathione reductase [41]. In an in vitro study, Pieniazek et al. [42] showed that IS may induce oxidative stress and decrease the antioxidant defense in mononuclear cells, leading to lipid and protein damage; thus evidencing the importance of maintaining the oxidative balance. Indeed, uremic toxins induce ROS production, which is related to oxidative stress and, therefore, to the inflammatory process [33,38]. Li et al. [43] evidenced that trimethylamine-N-oxide (TMAO) can increase the production of superoxide and proinflammatory cytokines while reducing eNOS activity in rats with CKD. The effect of IS on HUVECs was shown by Masai et al. [38], where the production of intracellular ROS (superoxide) was increased. This ROS activates transcription factors (e.g., NF-κB), therefore leading to the expression of inflammatory cytokines. Mitochondrial respiration is a potential source of ROS, as well as xanthine oxidase, NADPH oxidase, NO synthases, peroxidases, and other hemoproteins [44,45,46]. In HUVECs, IS leads to an increase in ROS production through the activation of NADPH oxidase, as well as causing mitochondrial dysfunction, thereby inducing cell death [44,45]. In contrast, ROS are related to the transduction of intracellular signals involved in a spectrum of biological processes [47]. ROS and their metabolites are involved in the activation of ions channel, such as transient receptor potential vanilloid 1 (TRPV1) and transient receptor potential ankyrin 1 (TRPA1), which are subfamilies of the transient receptor potential (TRP) channels [48,49,50]. Both TRPV1 and TRPA1 are involved in vascular tone and endothelial cell function regulation under physiological conditions [48,49]. Wang et al. [51] demonstrated that the activation of TRPV1 can suppress the inflammatory response of endothelial cells.

Furthermore, uremic toxins inhibit late stage autophagy, since the cells are more sensitive to oxidative stress in uremic conditions, contributing to endothelial dysfunction [52]. Inhibition or interference in the autophagic process in the endothelium by uremic toxins may lead to atherogenesis and arterial aging [52,53,54]. Taken together, these findings demonstrate that uremic toxins induce oxidative stress as well as the expression of proinflammatory molecules that are involved in the pathogenesis of CVD.

### 2.2. Uremic Toxins Contribute to the Prothrombotic State of the Endothelium

The endothelium is directly involved in the prothrombotic and antithrombotic balance of the hemostatic system. However, in uremic conditions endothelial function is compromised and may contribute to abnormal coagulation and fibrinolysis processes [55]. Prothrombotic properties can lead to the development of thrombotic disorders, such as thromboembolism and ischemia. In fact, clinical studies have reported that patients with CKD are at increased risk of thrombotic events [6,55,56,57]. In addition, CKD patients have enhanced levels of some coagulation and fibrinolysis factors, such as tissue factor (TF), von Willebrand factor (vWF), thrombomodulin, factor VIII, and D-dimer [55,58,59].

TF is a membrane protein in the coagulation cascade that initiates the extrinsic pathway of blood clotting in response to a vascular injury; however, under pathological conditions it can contribute to thrombus formation [60]. CKD patients have higher plasma levels of TF as well as greater TF procoagulant activity, which is observed in the shorter of lag time of thrombin generation in the plasma of CKD patients compared to healthy controls, suggesting a hypercoagulable state [58,61]. In vitro studies have shown that IS and IAA increased TF expression in endothelial cells, including HUVECs, HAECs, and cardiac-derived microvascular endothelial cells (HMVEC-C) [61,62]. Higher production of factor Xa by TF was also observed in HUVECs exposed to IS and IAA [61]. These data indicate that uremic toxins cause endothelial activation and procoagulant activity.

The endothelium produces vasoactive compounds relevant for the regulation of hemostasis, but this may be altered in uremic conditions. NO is an important vasodilator produced by eNOS in the endothelium that also inhibits platelet adhesion and aggregation [63]. In uremic conditions, however, NO production and bioavailability are reduced in endothelial cells [64,65]. In vitro studies have demonstrated that IS and inorganic phosphate decrease NO bioavailability, which is related, at least in part, to the increase in oxidative stress in endothelial cells [64,65,66]. Furthermore, patients with CKD have high levels of the uremic toxin asymmetric dimethylarginine (ADMA), which is an eNOS antagonist and, therefore, reduces NO production [67,68]. The endothelium also produces prostaglandins, such as prostaglandin E2 (PGE2), that have a procoagulant role in inducing platelet aggregation [69]. PGE2, derived from arachidonic acid, is produced by cyclooxygenase-2 (COX-2), which is overexpressed in endothelial cells exposed to IS and IAA [62,70]. Therefore, endothelial damage and inflammation contribute to the prothrombotic state. In vivo, greater thrombus formation was observed after vascular injury in rats treated with IS compared to those not exposed to toxin [71,72]. Based on these studies, the uremic toxins lead to a proinflammatory and prothrombotic endothelial phenotype, which is related to the occurrence of thrombotic events and other CVD in CKD patients.

Fibrinolysis is enhanced in CKD, as demonstrated by higher levels of tissue plasminogen activator (t-PA) and plasminogen activator inhibitor 1 (PAI-1) in this setting compared to healthy controls [58,73]. Likewise, there are also higher serum levels of D-dimer, a product of fibrin degradation, in CKD patients [55]. In vivo, Karbowska et al. [72] found increased levels of PAI-1, but not of D-dimer, in rats treated with IS compared to control group. Therefore, both coagulation and fibrinolysis systems are altered in CKD [58,73,74].

### 2.3. Uremic Toxins Increase Endothelial Permeability

Uremic toxins contribute to structural damage of the endothelial monolayer, which results in increased permeability. In CKD patients, studies have detected disruptions to the structure of the endothelial monolayer in renal arteries [13,75]. Loss of endothelial integrity and enhanced permeability are also seen in the aortic endothelium of nephrectomized rats, an animal model of CKD [76,77]. In vitro, PCS, IS, and uremic serum reduced the transendothelial electrical resistance (TEER), which indicates an increase in endothelial permeability [75,78,79]. In this regard, studies have also found that uremic toxins induced F-actin cytoskeletal remodeling in endothelial cells, leading to changes in cell morphology [13,79,80].

In vitro studies have shown that uremic toxins lead to the rupture of cell-cell junctions, especially related to vascular endothelial (VE)-cadherin and zonula occludens-1 (ZO-1) proteins [13,75,78,79]. VE-cadherin is a transmembrane protein that belongs to adherent junctions and plays a crucial role in cell–cell interaction [81,82]. ZO-1 is an intracellular protein that is part of tight junction proteins and regulates VE-cadherin [83]. Our group have demonstrated a decrease in the expression of VE-cadherin and ZO-1 in endothelial cells exposed to inorganic phosphate and uremic serum, while no change in expression was observed when cells were exposed to PCS and IS [13]. However, Chen et al. [75] recently demonstrated that PCS increased phosphorylation of VE-cadherin at tyrosine 658 (Y658) in HUVECs. In a similar manner, Assefa et al. [78] found that IS increased VE-cadherin phosphorylation in bovine aortic endothelial cells (BAECs). VE-cadherin phosphorylation is mediated by tyrosine kinases, such as Src, and is associated with enhanced endothelial permeability, possibly by inducing VE-cadherin internalization [81]. Based on this, Chen et al. [75] and Assefa et al. [78] observed that PCS and IS also increased Src phosphorylation. These findings suggest a Src-mediated VE-cadherin phosphorylation mechanism induced by uremic toxins, which results in loss of endothelial cell–cell interactions [75,78].

**Figure 2:**
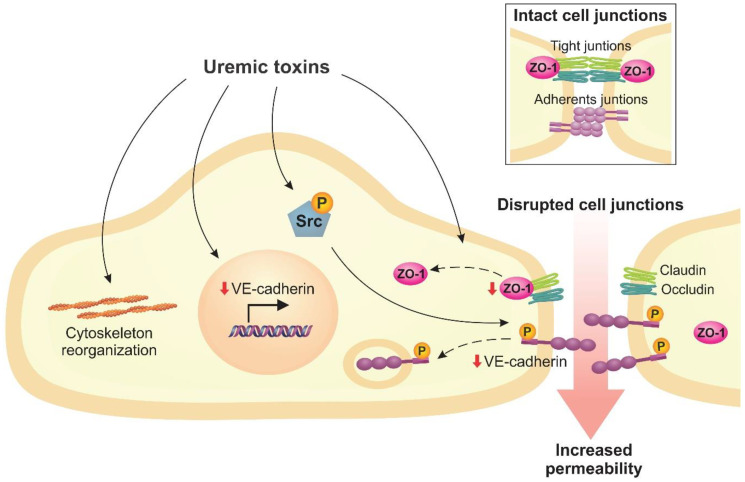
Uremic toxins lead to the loss of intercellular junctions, which results in increased endothelial permeability. This scheme demonstrates that uremic toxins induce cytoskeleton remodeling, reduce vascular endothelial (VE)-cadherin gene expression and activate Src, which phosphorylates VE-cadherin and leads to its internalization. In addition, uremic toxins also decrease zonula occludens-1 (ZO-1) protein levels.

The impairment of the endothelial barrier induced by uremic toxins contributes to the increased permeability that is associated with vascular injury and the development of CVD, such as atherosclerosis (Figure 2) [77,84]. However, studies have found that vitamin D supplementation attenuated the effects of uremic toxins on endothelial disruption in HUVECs, and in nephrectomized rats [76,79].

### 2.4. Uremic Toxins Impairs Endothelial Protective Mechanisms

Mechanisms that protect endothelial functions may be impaired by uremic toxins. One of these mechanisms is the repair capacity mediated by endothelial progenitor cells, which are recruited to injury sites for vascular regeneration [85,86,87]. Studies have demonstrated that uremic toxins cause dysfunction of endothelial progenitor cells, including decreased chemotactic motility and angiogenesis [85,86]. Furthermore, clinical studies have also shown a reduction in the number of endothelial progenitor cells in CKD patients [87,88,89].

The expression of molecules with protective functions in endothelial cells may be altered in uremic conditions. For example, Krüppel-like factor 2 (KLF2) expression, an important transcription factor for endothelial homeostasis, is suppressed by PCS, IS, AGEs, and uremic serum [90,91]. In a similar manner, IS and uremic serum reduce the expression of sirtuin 1 (SIRT1), which plays an important role in maintaining endothelial functions, inhibiting senescence and oxidative stress [72,91]. Moreover, Shang et al. [91] demonstrated that decreased expression of KLF2 and SIRT1 in endothelial cells exposed to uremic toxins is miR-92a-dependent. Uremic toxins can also modulate the Klotho expression, which has a protective role on the endothelium. In this regard, endothelial cells exposed to soluble Klotho had an increase in NO production [92]. Klotho also attenuated TNF-α-induced activation of NF-κB as well as the expression of ICAM-1 and VCAM-1 in endothelial cells [93]. Importantly, Klotho expression and its serum concentration are decreased in patients with CKD [94]. Interestingly, Sun et al. [95] demonstrated that PCS and IS are capable of reducing Klotho expression in renal tubular cells through an epigenetic mechanism, the DNA hypermethylation of its gene. Taken together, these data indicate that uremic toxins can negatively affect mechanisms that protect and repair the endothelium.

## 3. Transporters and Receptors of Uremic Toxins in the Endothelium

Transporters and receptors may change plasma levels of substrates through the secretion and reabsorption of many compounds, including uremic toxins [14,96,97]. In the endothelium, the expression of transporters and receptors of uremic toxins may play an important role in endothelial dysfunction. The organic anion transporter (OAT) family is a major class of transporters, which are essential for the elimination of many compounds and uremic toxins that are in anionic form at physiological pH. The 10 known isoforms of OATs belong to the family of solute carrier genes (SLC), more specifically solute carrier 22 (SLC22), and are well described in the literature [14,97]. OATs are located in various tissues and organs, including the liver, brain, kidney, and endothelium, where they mediate the movement of drugs and toxins between bodily fluid compartments and tissues [14,97,98,99].

Recently, we suggested that the uptake of IS and PCS are mediated by OATs (OAT1 and OAT3) in human endothelial cells. In this study, endothelial cells were treated with PCS and IS in the presence or absence of probenecid (an OAT inhibitor). After 60 min of treatment, both uremic toxins showed significant internalization, while no internalization was observed in the presence of probenecid, suggesting that OATs are involved in the transportation of these toxins [14]. However, more studies are necessary to provide robustness to these results. Other studies have shown the uptake of uremic toxins by OATs, such as Miyamoto et al. [100], who demonstrated the uptake of PCS by OAT3 in HK-2 cells, and Deguchi et al. [101], who described the uptake of IS, IAA, and hippurate using stable transfectants of rOat1/hOAT1 and rOat3/hOAT3 in LLC-PK1 cells, indicating the contribution of OAT1 and OAT3 to the uptake of IS, and of OAT1 to the uptake of IAA and hippurate.

The organic anion transporting polypeptides (OATPs) are a different category of organic anion transporters involved in the uptake of uremic toxins in the endothelium. OATPs belong to the SLCO superfamily and they have been detected in various tissues, such as lung, liver, kidney, and brain [102,103]. Grube et al. [104] showed that these transporters are expressed in the cardiac endothelium. Also, Bronger et al. [105] demonstrated the OATPs expression in the luminal membrane of the blood–brain barrier endothelial cells. Nakano et al. [106] observed that OATP2B1 expressed by human macrophages are involved in the in vitro IS uptake; thus, inducing vascular inflammation. It has also been showed that there are transporters that efflux uremic toxins, such as multidrug resistance-associated proteins (MRPs) [107,108,109].

Transporter-mediated phosphate uptake may be related to the endothelial dysfunction caused by hyperphosphatemia in patients with CKD [36,110]. The sodium-dependent phosphate cotransporters (NaPiTs) are composed of type-I (SLC17 family), type-II (SLC34 family), and type-III (SLC20 family). The two known type-III NaPiTs—PiT-1 and PiT-2—are involved in phosphate homeostasis in the human body, contributing to cellular uptake [111,112]. Inden et al. [112] demonstrated that PiT-2 is present in neurons, astrocytes, and vascular endothelial cells. Abbasian et al. [113] also observed an increase in the intracellular concentration of inorganic phosphate in endothelial cells exposed to hyperphosphatemia conditions, which was reversed by PiT-1 knockout and inhibition. The effect of phosphate on vascular function has been described by Six et al. [36] using in vitro (HUVECs and HVSMCs) and in vivo (mouse model) assays, where it was shown that increased levels of phosphate in blood can lead to oxidative stress, causing deleterious effects on vascular function and structure. In patients, higher mortality during the pre-dialysis phase and acceleration of kidney function decline have been shown to be correlated with hyperphosphatemia [110,114].

Advanced glycation end products (AGEs), proinflammatory and pro-oxidative compounds classified as uremic toxins, are produced through the non-enzymatic glycation and oxidation of proteins, lipids, and nucleic acids. Their accumulation in CKD promotes endothelial dysfunction and subsequent diseases [115,116,117,118,119,120]. Atherosclerosis, autoimmune disease, diabetes mellitus, and inflammatory diseases are closely linked to the AGE receptor-ligand axis [121,122,123,124]. AGEs induce cell injury by interacting with their receptor, denominated receptor for advanced glycation end products (RAGE), which is a ubiquitous multiligand transmembrane cell surface receptor of the immunoglobulin superfamily [116,125,126,127]. We also previously demonstrated that AGEs induced RAGE expression in endothelial cells [128]. The importance of blocking RAGE is described by Wang et al. [124], who used in vivo (rat model) and in vitro (HAECs) assays to emphasize that the inhibition of interaction between AGEs and RAGE helps to soften endothelial dysfunction. Thus, blocking the RAGE-ligand axis may be a therapeutic target, agreeing with other studies [116,121,123,129,130,131,132].

PCS and IS may also activate epidermal growth factor receptor (EGFR), transmembrane protein with cytoplasmic kinase activity with role in signaling transduction [133]. In vitro analyzes demonstrated that PCS and IS induced the dimerization and activation of EGFR by phosphorylation [133,134]. IS also enhanced EGFR expression in VSMCs [134]. Consequently, PCS and IS led to increased expression of matrix metalloproteinases 2 and 9 (MMP2 and MMP9) in renal cells through the EGFR signaling pathway [133].

Taken all together, the experimental data available indicates that the interactions between a transporter or receptor and their ligands (e.g., uremic toxins) are important targets for therapeutic strategies, since these interactions trigger deleterious effects in the endothelium and in other body tissues. However, more studies are needed to better understand the mechanisms involved in this process.

## 4. Cell Signaling Pathways Altered by Uremic Toxins

Uremic toxins lead to phenotypic endothelial changes through the activation of cellular signaling pathways. Given the complexity of these pathways, important proteins that modulate the cellular response to uremic toxins stand out, such as AhR, NF-κB, and MAPKs. These molecules, therefore, have potential to be used as therapeutic targets in CKD.

### 4.1. AhR Pathway

AhR is a ligand-inducible transcription factor that belongs to the basic helix–loop–helix transcription factor family. Inactivated AhR is found in the cytoplasm bound to chaperones, which dissociate when AhR is activated by binding to a ligand [135]. Studies have identified several exogenous and endogenous ligands for AhR, such as 2,3,7,8-tetrachlorodibenzo-p-dioxin (TCDD), dioxin-like planar polychlorinated biphenyls (PCBs), and uremic toxins, including IS, IAA, indoxyl glucuronide, kynurenic acid, and other tryptophan derivatives [62,135,136,137,138,139,140]. Activated AhR can induce genomic signaling, known as the canonical pathway, as well as the non-genomic pathway.

In the genomic signaling pathway, activated AhR is translocated to the nucleus, where it forms a dimer with aryl hydrocarbon receptor nuclear translocator (ARNT) [135]. The AhR/ARNT complex binds to the xenobiotic responsive element (XRE; 5′-GCGTG-3′) in the promoter region of several target genes, inducing their expression [135,141]. Studies have shown that IS and IAA induce AhR translocation to the nucleus in endothelial cells [26,62,136]. In addition, overexpression of *CYP1A1* and *CYP1B1*, genes directly regulated by AhR/ARNT, was found in endothelial cells exposed to IS and IAA, an effect that was reversed with AhR inhibitors [26,62,70,136]. Furthermore, it has been demonstrated in HUVECs that IS and IAA, through AhR, induce AhRR expression, an AhR repressor that competes for ARNT binding, which ultimately leads to a negative regulatory loop for AhR [61,70,141].

In the non-genomic pathway, activated AhR interacts with various other signaling molecules, such as NF-κB and Src, independently of ARNT [62]. Ito et al. [26] demonstrated that IS increased the expression of E-selectin in an AhR-dependent manner in HUVECs. Despite that, the authors verified that AhR did not directly bind to the E-selectin gene promoter [26]. However, it was found that E-selectin overexpression was associated with the activity of the transcription factor activator protein-1 (AP-1), which is induced by AhR through the non-genomic pathway [26]. Similarly, Addi et al. [62] demonstrated that HUVECs exposed to IAA had an increase in TF gene expression, but AhR was not linked to the gene promoter despite the effect being reversed with AhR knockout and inhibition. The authors then found that NF-κB was essential for increasing TF expression, but its activity was decreased by the AhR inhibitor, suggesting a regulation between them [62].

Studies have shown that the activation of AhR by uremic toxins is involved in vascular inflammation, permeability, and the development of CVD [26,78,136]. In HUVECs, IS induced MCP-1 and E-selectin expression, proinflammatory molecules involved in leukocyte recruitment and adhesion to the endothelium, in an AhR-dependent manner [26,136]. In vivo, Ito et al. [26] also found that IS increased interaction between leukocytes and the endothelium of the femoral artery in wild-type mice, an effect that was not seen in endothelial cell-specific AhR knockout mice treated with IS. Furthermore, the activation of AhR by IS also enhanced Src phosphorylation and, consequently, VE-cadherin phosphorylation, inducing an increase in endothelial permeability that was reversed with AhR inhibitors [78]. Koizumi et al. [39] demonstrated that IS-induced senescence of HUVECs is AhR-dependent and may contribute to CVD. In HUVECs, IAA and IS increased the expression of TF through AhR, which is associated with the pathogenesis of atherosclerosis and thrombosis [61,62]. Therefore, these studies suggest that AhR activation induced by uremic toxins has an important role in endothelial dysfunction and vascular injury. Interestingly, IS through the AhR pathway led to upregulation of OAT1 in renal proximal tubule cells as well as P-glycoprotein, an efflux pump that is part of the ABC transporter superfamily, in human hepatoma cells [142,143]. These data suggest that the AhR pathway may be involved in the regulation of the expression of cellular transporters.

AhR activating potential (AhR-AP) corresponds to the combination of all AhR agonists present in uremic serum, such as IS, IAA, and indoxyl glucuronide. Dou et al. [144] demonstrated that uremic serum from stage 3 to 5 and stage 5D CKD patients had higher AhR-AP than serum from healthy controls by an AhR-responsive bioassay. In addition, the authors reported that AHR-AP is associated with cardiovascular events in CKD patients [144]. In vivo, Dou et al. [144] detected higher serum levels of AhR agonists as well as overexpression of *Cyp1a1*, a gene regulated by AhR, in the aorta and heart of nephrectomized mice compared to AhR^-/-^ nephrectomized or wild-type mice. In a cohort of patients with ESRD, Shivanna et al. [145] noted greater AhR activity in uremic serum compared to that of healthy controls. Kolachalama et al. [140] also found increased AhR activity and TF levels in serum from CKD patients who had thrombotic events compared to their counterparts without thrombosis. Taken together, these data indicate a relationship between uremic toxins, AhR activation, and the development of CVD [140,144,146].

### 4.2. NF-κB Pathway

NF-κB is a family of transcription factors that play a crucial role in endothelial inflammation. There are five forms of NF-κB proteins: p50 (NF-κB1), p52 (NF-κB2), p65 (RelA), RelB, and c-Rel. The p52 and p50 proteins are derived from the p100 and p105 forms, respectively. NF-κB family members form dimers, of which the p50/p65 dimer is the most common [147,148]. In the inactivated state, NF-κB dimers are found in the cytoplasm bound to inhibitory proteins, such as IκBα and IκBβ. In the presence of a stimulus, NF-κB dissociates from the inhibitory proteins, which are degraded. In the canonical pathway, the activation of IκB kinase (IKK) mediates the phosphorylation of IκBα, releasing NF-κB (mainly the p50, p65, and c-Rel forms) which is then translocated to the nucleus [147,148]. On the other hand, in the non-canonical pathway, also known as the alternative pathway, the activation of NF-κB-inducing kinase (NIK) leads to p100 phosphorylation and processing to the p52 form, which is translocated to the nucleus along with RelB [147,148].

Studies have shown that the NF-κB pathway participates in gene regulation in endothelial cells in the uremic conditions [38,70,149]. HUVECs exposed to uremic serum had greater IκB degradation and, consequently, higher levels of p50/p65 in the nucleus compared to those exposed to healthy serum [150]. In HUVECs, Tumur et al. [149] demonstrated that IS induced p65 phosphorylation as well as MCP-1 and ICAM-1 overexpression, which was reversed with NF-κB inhibitors. Furthermore, Masai et al. [38] reported an increase in the translocation of p65 to the nucleus and MCP-1 upregulation in HUVECs exposed to IS. Interestingly, the authors verified that this effect was suppressed by ERK1/2 and p38 MAPK inhibitors, indicating the participation of these proteins in the activation of the IS-induced NF-κB pathway [38]. In addition, studies have demonstrated an increase in the translocation of p50 and p65 to the nucleus in HUVECs exposed to IAA, which was decreased with AhR and p38 MAPK inhibitors [62,70]. Inhibition of NF-κB also reversed IAA-induced COX-2 and TF overexpression in HUVECs [62,70]. Based on these data, it is suggested that the NF-κB pathway is important for the upregulation of proinflammatory proteins in uremic conditions.

### 4.3. MAPK Pathway

MAPK are a family of serine/threonine kinases that are activated by phosphorylation [113]. There are three main groups of MAPK: Extracellular signal-regulated kinase (ERK1 and ERK2), C-Jun N-terminal kinase (JNK1, JNK2, and JNK3), and p38 MAPKs (α, β, γ, and δ) [151]. The MAPK signaling pathway is activated in the presence of a stimulus and, consequently, mediates the cellular response through activation of other proteins, such as transcription factors, by phosphorylation [152,153]. Therefore, MAPK has a significant role in signal transduction [151].

**Figure 3:**
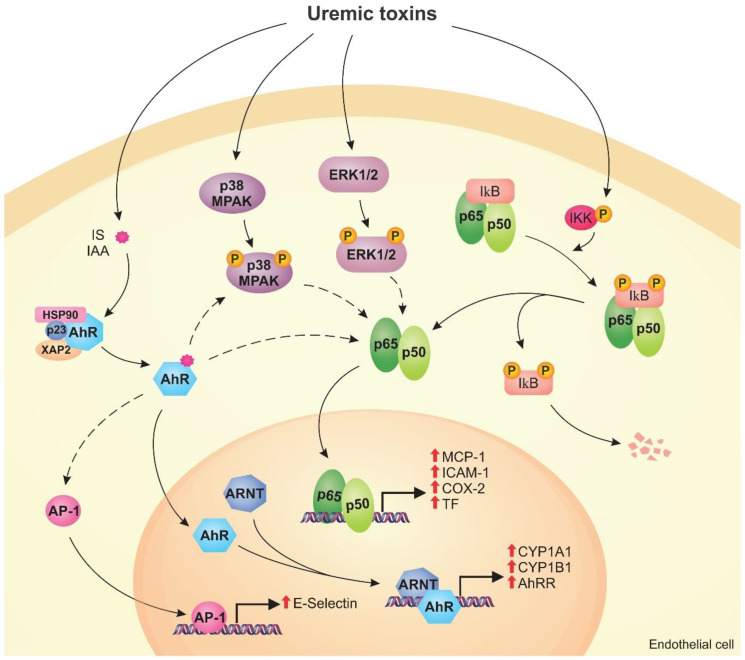
Uremic toxins activate the aryl hydrocarbon receptor (AhR), nuclear factor kappa B (NF-κB), and mitogen-activated protein kinase (MAPK) pathways in endothelial cells. Toxins such as indoxyl sulfate (IS) and indole-3 acetic acid (IAA) activate AhR, which is translocated to the nucleus, forms a dimer with aryl hydrocarbon receptor nuclear translocator (ARNT), and induces the expression of CYP1A1, CYP1B1, and AhRR (genomic pathway). Activated AhR can stimulate other pathways (non-genomic pathway), including the activation of AP-1 that induces E-selectin expression. Uremic toxins also cause the activation of the MAPK pathway, such as p38MAPK and ERK1/2, by phosphorylation. MAPK and AhR can induce the NF-κB pathway. In uremic conditions, IκB is degraded and p50/p65 are translocated to the nucleus, inducing monocyte chemoattractant protein-1 (MCP-1), intercellular adhesion molecule-1 (ICAM-1), cyclooxygenase-2 (COX-2), and tissue factor (TF) expression. The activation of these pathways by uremic toxins can lead to endothelial dysfunction.

In uremic conditions, studies have shown that the MAPK pathway is activated in endothelial cells. Uremic serum enhanced ERK1/2 phosphorylation in HUVECs compared to normal serum [150]. IAA and IS also induced phosphorylation of p38 MAPK and ERK1/2 in HUVECs in the first 30 min of exposure [38,70]. In addition, MAPK pathway inhibition can reverse the effects of uremic toxins [38,62,70]. Inhibition of p38 MAPK reduced IAA-induced TF and COX-2 overexpression in HUVECs, the expression of which are possibly modulated by the AhR/p38MAPK/NF-κB pathway [62,70]. In another study, it was shown that inhibition of ERK1/2 and p38 MAPK suppressed IS-induced p65 phosphorylation (NF-κB) as well as MCP-1 overexpression in HUVECs [38]. Figure 3 shows the mechanisms modulated by the AhR, NF-κB, and MAPK signaling pathways in endothelial cells exposed to uremic toxins.

## 5. Modulation of MicroRNAs by Uremic Toxins in Endothelial Cells

MicroRNAs (miRNAs) play an important role in the regulation of endothelial cell function via the modulation of eNOS-derived nitric oxide bioavailability, angiogenesis, and innate immune response [91]. Recent studies have demonstrated the involvement of miRNAs in endothelial dysfunction since they act as regulators in endothelial cells [154,155,156]. miRNAs are part of a family of small endogenous noncoding RNA made up of about 21 nucleotides, and influence physiological/pathological processes, including cell growth, differentiation, and apoptosis [91,156]. Uremic toxins can upregulate miRNA-92a, as well as miR-142-3p, miRNA-92a-3p, and miRNA-489-3p, suppressing the expression of genes critical for endothelial homeostasis, thus contributing to their dysfunction [91,156,157]. Uremic toxins can also cause the downregulation of miRNAs, such as lower levels of miRNA-214, which promotes apoptosis [158]. In patients with CKD, lower levels of circulating miR-126 and miR-223 were associated with a lower estimated glomerular filtration rate and with higher mortality and cardiovascular events [159]. These mechanisms may lead to the discovery of a new perspective for the treatment of CKD [160,161]. All these data suggest possible new targets for the treatment of endothelial injury in CKD, demonstrating the importance of studies of the other effects of uremic toxins on endothelial dysfunction.

## 6. Uremic Toxins Induce the Formation of Endothelial Microparticles

Endothelial microparticles (EMPs) are vesicles derived from the cell membrane, between 0.1 µm and 1 µm in size, which carry content from the source cell, including proteins and miRNAs [162,163]. EMPs interact directly with target cells, such as VSMCs, monocytes, endothelial progenitor cells, and other endothelial cells [163,164,165]. This interaction leads to the internalization of EMPs and the transfer of their active biomolecules to the target cell, which may activate signaling pathways [164]. Therefore, EMPs are important tools of intercellular communication formed in physiological and pathological processes, including in CKD [162,166].

In uremic conditions, it was demonstrated that PCS, IS, and inorganic phosphate induced the formation of EMPs from endothelial cells [113,163,167,168,169]. Studies have also shown that EMPs are related to endothelial dysfunction in CKD patients [17,167,170,171]. In vitro, Carmona et al. [163] showed that EMPs from endothelial cells exposed to IS had increased levels of ICAM-1 and PECAM-1, and miRNAs (e.g., miR-181a-5p, miR-4454, and miR-150-5p). In the same study, the authors found that IS-induced EMPs had an anti-angiogenic effect on endothelial progenitor cells, which are important in the endothelium repair process [163]. Furthermore, IS- and IAA-induced EMPs had greater procoagulant activity, due to the production of factor Xa by TF, compared to EMPs from cells not exposed to these toxins [61]. Similarly, EMPs from cells exposed to hyperphosphatemia conditions caused an increase in thrombin formation, also indicating procoagulant activity [113]. Soriano et al. [165] isolated EMPs from CKD patients and found that they increase osteocalcin (OCN) expression in endothelial progenitor cells, VSMCs, and fibroblasts, which indicates cellular dysfunction and vascular calcification. Furthermore, IS-induced EMPs enhanced the proliferation of VSMCs in vitro and in an ex vivo model, effects that contribute to neointimal hyperplasia [164,172]. These findings suggest that EMPs formed in the uremic conditions can mediate the dysfunction of target cells and contribute to vascular damage and the development of CVD.

## 7. Therapeutic Strategies for Uremic Toxins

Endothelial dysfunction is considered one of the most important triggers and mainstays of CVD in CKD patients. As uremic toxins may lead to endothelial dysfunction by multiple pathways, several pre-clinical and clinical studies have investigated whether therapeutic strategies devoted to reducing uremic toxin levels could improve vascular health.

As the guts are the main source of uremic toxins, approaches targeting the gut microbiome have been used to decrease intestinal generation of those compounds. In this regard, the short-term administration of prebiotics, probiotics and symbiotics to CKD patients may favorably modify the microbiome and lower the serum levels of uremic toxins, such as IS and PCS [173,174,175,176]. Changing the source of protein intake from animal-based to plant-based diet might be other strategy to reduce intestinal production of uremic toxins. For instance, it has been reported that patients on hemodiafiltration (HDF) eating a vegetarian diet present lower plasma levels of IS and PCS [177]. Other potential advantage of vegetarian diet is a better control of serum phosphate levels [177,178]. It should be kept in mind that, to date, high-quality intervention trials dedicated to modulate intestinal production of uremic toxins are still scarce.

AST-120 is an oral carbon adsorbent that can bind to IS and PCS precursors in the gut lumen, therefore lowering serum levels of these toxins [179]. AST-120 treatment of CKD mice improved vascular function, reduced aortic VCAM-1 and ICAM-1 expression, and prevented an increase in pulse wave velocity [35]. Administration of AST-120 to CKD patients (n = 40) for 6 months resulted in a significant increase in flow-mediated dilation (FMD), an early marker of endothelial dysfunction, with a decrease in IS levels and oxidative stress [180]. It has also been reported that sevelamer, a non-absorbable phosphate binder, may improve endothelial function. Its administration for a short period of 8 weeks to stage 4 CKD patients improved FMD, an effect also observed for calcium acetate [181,182]. Sevelamer treatment in CKD mice decreased serum phosphate levels and ameliorated cardiovascular abnormalities, including pulse-wave velocity [183]. This beneficial effect of sevelamer on endothelial function might be further explained by its pleiotropic effects, such as binding to AGEs [128]. Interestingly, the vitamin D receptor activator paricalcitol, despite not targeting uremic toxins, may ameliorate endothelial function in moderate CKD [184]. In vitro studies have demonstrated that paricalcitol may protect endothelial cells from uremic toxin exposure by promoting the expression of VE-cadherin at intercellular junctions, which ultimately leads to the recovery of endothelial barrier function and cell–cell interactions [79]. In addition, in CKD patients, there is a decrease in active vitamin D levels (25 hydroxyvitamin D and 1,25-dihydroxyvitamin D), that plays an important role in the metabolism of calcium, phosphate, and parathyroid hormone (PTH) [185].

Evidences indicates that HDF is superior to conventional hemodialysis therapy for recovery of endothelial function, likely due to a broader clearance of uremic toxins [186,187]. Despite of similar removal of protein-bound uremic toxins, HDF provides superior removal of low-molecular weight protein when compared to high-flux-hemodialysis [188,189]. Among the three main options for convective dialysis therapies, post-dilution HDF has been reported to be more effective than pre-dilution HDF and pre-dilution hemofiltration for the removal of uremic toxins [190]. Interestingly, a recent study has reported that use of novel medium cut-off dialyzers in hemodialysis therapy may improve the removal of larger middle molecules compared to high-flux hemodialysis and HDF [191].

Due to the limited removal of protein-bound uremic toxins by conventional dialysis technique, new approaches based on adsorption-based techniques and on displacement of these toxins from their albumin binding sites, in order to increase their dialysable free-fraction, have been examined. In a proof-of-concept study, Madero et al. [192] have elegantly demonstrated that the infusion of ibuprofen, used as a competitive binding inhibitor, during a conventional 4-h high-flux hemodialysis treatment led to greater dialytical removal and serum levels reduction of IS and PCS. A recent in vitro study has reported promising results for the use of adsorber techniques to enhance hydrophobic uremic toxins removal, without harmful effects on hemocompatibility [193].

Finally, as uremic toxins levels has been related to residual kidney function in dialysis patients [189,194], its preservation is ultimately an important therapeutic approach for a better control of uremic toxins levels. Even though these results are promising, they should be interpreted with caution. Most studies are of limited duration, have included a low number of patients, and have been based on surrogate markers rather than on hard outcomes; this prevents the assumption that these effects on endothelial function will be translated into clinical benefits, such as lower rates of cardiovascular events. Furthermore, an in vitro effect is not a guarantee of an equivalent action in the patient.

## 8. Final Considerations

**Figure 4:**
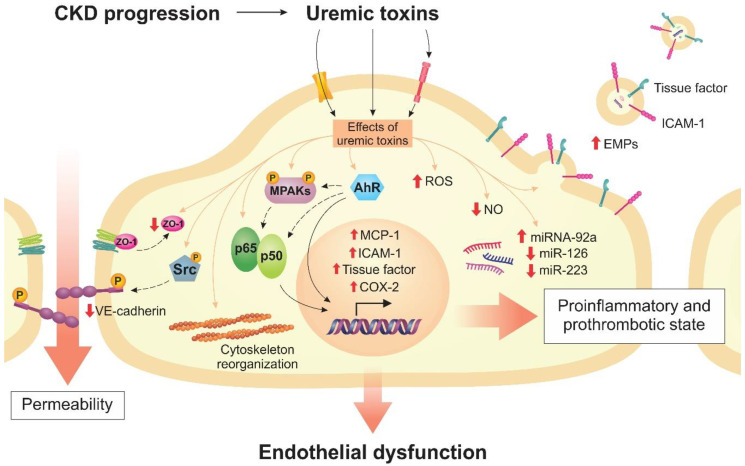
Uremic toxins cause endothelial dysfunction. Transporters and receptors mediate the interaction between endothelial cells and uremic toxins, with subsequent activation of signaling pathways, expression of proinflammatory and prothrombotic molecules, increase in reactive oxygen species (ROS), decrease in nitric oxide (NO), modulation of miRNAs, cytoskeleton remodeling, formation of endothelial microparticles (EMPs), and loss of cell–cell junctions.

Uremic toxins, when in high concentrations in the bloodstream, play an important role in endothelial dysfunction. These compounds can lead to changes in vascular homeostasis and cell function, including changes in cell structure, the expression of receptors and transporters, and the activation of signaling pathways, leading to the release of cytokines and chemokines and the beginning of the inflammatory process. In this review, we described the effects of uremic toxins in endothelial permeability—mainly their relationship with VE-cadherin and ZO-1 proteins—and their possible transporters and receptors. We also described cell signaling pathways, specifically the AhR, NF-κB, and MAPK pathways, and the inflammation and thrombosis processes, as well as EMPs formation and the involvement of uremic toxins in epigenetic regulation through miRNAs (Figure 4). The available experimental data demonstrated the importance of studying the interaction between uremic toxins and endothelium, evidencing new therapeutic strategies.
